# A short, animated video to improve good COVID-19 hygiene practices: a structured summary of a study protocol for a randomized controlled trial

**DOI:** 10.1186/s13063-020-04449-1

**Published:** 2020-06-03

**Authors:** Alain Vandormael, Maya Adam, Merlin Greuel, Till Bärnighausen

**Affiliations:** 1 Heidelberg Institute of Global Health, University of Heidelberg, Heidelberg, Germany; 2 KwaZulu-Natal Research and Innovation Sequencing Platform, University of KwaZulu-Natal, Durban, South Africa; 3 Department of Pediatrics, Stanford University School of Medicine, Stanford, CA USA; 4 Department of Global Health and Population, Harvard T.H Chan School of Public Health, Boston, MA USA; 5 Africa Health Research Institute (AHRI), Somkhele, KwaZulu-Natal South Africa

**Keywords:** COVID-19, randomised controlled trial, protocol, entertainment-education, behavioral intent, knowledge, list experiment

## Abstract

**Objectives:**

Entertainment-education (E-E) media can improve behavioral intent toward health-related practices. In the era of COVID-19, millions of people can be reached by E-E media without requiring any physical contact. We have designed a short, wordless, animated video about COVID-19 hygiene practices—such as social distancing and frequent hand washing—that can be rapidly distributed through social media channels to a global audience. The E-E video’s effectiveness, however, remains unclear.

The study aims to achieve the following objectives. To:

1. Quantify people’s interest in watching a short, animated video about COVID-19 hygiene (abbreviated to CoVideo).
2. Establish the CoVideo’s effectiveness in increasing behavioural intent toward COVID-19 hygiene.
3. Establish the CoVideo’s effectiveness in improving COVID-19 hygiene knowledge.

**Trial Design:**

The present study is a multi-site, parallel group, randomized controlled trial (RCT) comparing the effectiveness of the CoVideo against an attention placebo control (APC) video or no video. The trial has an intervention arm (CoVideo), placebo arm (APC), and control arm (no video). Nested in each trial arm is a list experiment and questionnaire survey, with the following ordering. *Arm 1*: the CoVideo, list experiment, and questionnaire survey. *Arm 2*: the APC video, list experiment, questionnaire survey, and CoVideo. *Arm 3*: the list experiment, questionnaire survey, and CoVideo. For each list experiment, participants will be randomized to a control or treatment group. The control group will receive a list of five items and the treatment group will receive the same five items plus one item about COVID-19 hygiene. We will use the list experiment to reduce response bias associated with socially desirable answers to COVID-19 questions. The questionnaire survey will include items about the participant’s age, sex, country of residence, highest education, and knowledge of COVID-19 spread. After completing the list experiment and questionnaire survey, participants in Arms 2 and 3 will receive the CoVideo to ensure post-trial access to treatment.

**Participants:**

This will be an online study setting. We will use Prolific Academic (ProA: https://www.prolific.co) to recruit participants and host our study on the Gorilla™ platform (www.gorilla.sc). To be eligible, participants must be between the age of 18 and 59 years (male, female, or other) and have current residence in the United States, the United Kingdom, Germany, Spain, Mexico, or France. Participants will be excluded from the study if they cannot speak English, German, French, or Spanish (since the instructions and survey questions will be available in these 4 languages only).

**Intervention and comparator:**

The intervention is an E-E video about COVID-19 hygiene (CoVideo). Developed by our co-author (MA) for Stanford Medicine, the CoVideo is animated with sound effects, and has no words, speech, or text. The CoVideo shows how the novel coronavirus is spread (airborne, physical contact) and summarizes the public’s response to the COVID-19 outbreak. Key components of the CoVideo are the promotion of five hygiene practices: i) social distancing and avoiding group gatherings, ii) frequently washing hands with soap and water or sanitizer, iii) cleaning surfaces at home (e.g., kitchen counters), iv) not sharing eating utensils, and v) avoidance of stockpiling essential goods (such as toilet paper and face masks). The CoVideo, which was designed for universal reach and optimized for release on social media channels, can be viewed at https://www.youtube.com/watch?v=rAj38E7vrS8.

The comparators are an APC video (Arm 2) or no video (Arm 3). The APC video is similar in style to the CoVideo; it is also animated with a duration of 2.30 minutes, has sound effects but no words, speech, or text. The video message is about how small choices become actions, which become habits, which become a way of life. It is available at https://www.youtube.com/watch?v=_HEnohs6yYw. Each list experiment will have a control list as the comparator. The control list is needed to measure the prevalence of behavioral intent toward COVID-19 hygiene.

**Main outcomes:**

This study will measure primary and secondary outcomes related to COVID-19 hygiene. By hygiene, we mean the adoption of behaviors or practices that reduce the chances of being infected or spreading COVID-19. As our primary outcome, we will measure changes in behavioral intent toward five hygiene practices: social distancing, washing hands, cleaning household surfaces, not sharing eating utensils, and not stockpiling essential goods. As a secondary outcome, we will measure knowledge about behaviors that can prevent the spread of COVID-19.

**Randomization:**

Using a web-based randomization algorithm, Gorilla will randomly allocate participants to the intervention (CoVideo), placebo (APC), or control (no video) arm (sequence generation) at a 1:1:1 ratio. Within each trial arm, Gorilla will randomly allocate participants at a 1:1 ratio to the control or treatment group. Items in the lists will be randomly ordered to avoid order effects. The presentation order of the list experiments will also be randomized.

**Blinding:**

Because ProA handles the interaction between the study investigators and participants, the participants will be completely anonymous to the study investigators. The outcome measures will be self-reported and submitted anonymously. All persons in the study team will be blinded to the group allocation.

**Numbers to be randomized:**

The Gorilla algorithm will randomize 6,700 participants to each trial arm, giving a total sample size of 20,100.

**Trial status:**

The protocol version number is 1.0 and the date is 18 May 2020. Recruitment is expected to end by 22 June 2020. Thus far, the study investigators have recruited 2,500 participants on ProA. Of these participants, 800 have completed the study on the Gorilla platform.

**Trial registration:**

The study and its outcomes were registered at the German Clinical Trials Register **(**www.drks.de**)** on May 12^th^, 2020, protocol number: #DRKS00021582. The study was registered before any data was collected.

**Full protocol:**

The full protocol is attached as an additional file, accessible from the Trials website (Additional file 1). In the interest in expediting dissemination of this material, the familiar formatting has been eliminated; this Letter serves as a summary of the key elements of the full protocol.

## Supplementary information

**Additional file 1.** Full Protocol.

## Data Availability

Data will be collected and stored on the Gorilla platform. The study investigators own and have complete control of the research data, which can be accessed at any time. For statistical analysis, the data will be downloaded and safely stored on a computing system maintained by the University of Heidelberg.

