# Supplementary material for: A short, animated video to improve good COVID-19 hygiene practices: a structured summary of a study protocol for a randomized controlled trial
Source: Trials. 2020 Jun 3;21:469. doi: 10.1186/s13063-020-04449-1 (PMC7267760; doi:10.1186/s13063-020-04449-1)
Supplement: Supplementary file 1 — Additional file 1. Full Protocol. [file 13063_2020_4449_MOESM1_ESM.docx]

**A short, animated video to improve good COVID-19 hygiene practices: study protocol for a multi-site randomized controlled trial**

Alain Vandormael^1,2^, Maya Adam^3^, Merlin Greuel^1^, Till Bärnighausen^1,4,5^

^1^ Heidelberg Institute of Global Health, University of Heidelberg, Heidelberg, Germany

^2^ KwaZulu-Natal Research and Innovation Sequencing Platform, University of KwaZulu-Natal, Durban, South Africa

^3^ Department of Pediatrics, Stanford University School of Medicine, Stanford, CA, USA

^4^ Department of Global Health and Population, Harvard T.H Chan School of Public Health, Boston, USA.

^5^ Africa Health Research Institute (AHRI), Somkhele, KwaZulu-Natal, South Africa

## Administrative information

| Title | A short, animated video to improve good COVID-19 hygiene practices: study protocol for a multi-site randomized controlled trial |
| --- | --- |
| Trial registration | The study and its outcomes were registered at the German Clinical Trials Register ([www.drks.de](http://www.drks.de)) on May 12^th^, 2020: #DRKS00021582. |
| Protocol version | 1.0, 18 May 2020. |
| Funding | This study is funded by an Alexander von Humboldt University Professor Prize awarded to Dr. Till Bärnighausen. |
| Author details | 1 Heidelberg Institute of Global Health, University of Heidelberg, Heidelberg, Germany2 KwaZulu-Natal Research and Innovation Sequencing Platform, University of KwaZulu-Natal, Durban, South Africa3 Department of Pediatrics, Stanford University School of Medicine, Stanford, CA, USA4 Department of Global Health and Population, Harvard T.H Chan School of Public Health, Boston, USA.5 Africa Health Research Institute (AHRI), Somkhele, KwaZulu-Natal, South Africa |
| Name and contact information for the trial sponsor | Not applicable |
| Role of sponsor | Not applicable |

Abstract

Background: Entertainment-education (E-E) media can improve behavioral intent toward health-related practices. In the era of COVID-19, millions of people can be reached by E-E media without requiring any physical contact. We have designed a short, wordless, animated video about COVID-19 hygiene practices—such as social distancing and frequent hand washing—that can be rapidly distributed through social media channels to a global audience. The E-E video’s effectiveness, however, remains unclear.

**Methods/design:** This is a multi-site, parallel group, randomized controlled trial comparing the effectiveness of an E-E video on COVID-19 hygiene against (i) an attention placebo control (APC) video and (ii) no video. As our primary outcome, we will use a list randomization approach to measure behavioral intent toward COVID-19 hygiene. In each trial arm, participants will be randomized to a control list or a control list plus an item about social distancing, washing hands, cleaning household surfaces, sharing of eating utensils, and the stockpiling of essential goods. As a secondary outcome, we will measure knowledge about behaviors that can prevent the spread of COVID**-**19. We will leverage a regression framework to analyse the primary and secondary end-points. Using an online platform, we will recruit 20,100 participants (aged 18–59 years) from the United States of America, the United Kingdom, Germany, Spain, France, and Mexico.

**Discussion:** This trial will utilize several randomization procedures, list experimentation methods, and state-of-the-art online technology to demonstrate the effectiveness of an E-E video to improve COVID-19 hygiene uptake. Our results will inform future E-E video campaigns for COVID-19 hygiene and similar public health intervention needs.

Keywo**rds:** COVID-19, randomised controlled trial, protocol, entertainment-education, behavioral intent, knowledge, list experiment.

# Background and rationale

A large amount of information about novel coronavirus (COVID-19) hygiene has been disseminated by the traditional mass media since the outbreak of the pandemic.^1–3^ However, it is not clear if this dissemination has improved behavioral intent to practice COVID-19 hygiene. By hygiene, we mean the public’s adoption of practices, such as social distancing, reduced physical contact, and hand/surface sanitization (among others), to reduce the spread of COVID-19.

Arguably, one possible limitation of mainstream mass media is that it has been perceived as politicized and culturally localized, thus limiting the persuasiveness of COVID-19 hygiene messages.^4,5^ It is also likely that traditional mass media channels (e.g., local or national television networks) have missed key segments of the population, such as young people (25–40 years) who are disproportionately more likely to transmit the virus to older people ($\geq$ 60 years).^6^ An effective public health response could therefore benefit from focused entertainment-education (E-E) approaches that increase behavioral intent to take-up COVID-19 hygiene.^7^

To improve behavioral intent toward COVID-19 hygiene, we have designed an animated, wordless, video (abbreviated to CoVideo) that can be rapidly distributed to a diverse and global audience through social media channels. With a short duration (approximately 2.30 minutes), the CoVideo contains no speech and minimizes cultural signifiers to increase universality and appeal. The CoVideo was released on Stanford Medicine’s YouTube channel on March 21, 2020, and went viral within 24 hours. After ten days, it had reached 332,000 views on YouTube, 220,000 views on Instagram, 294,000 views on Facebook, and 402,000 views on Twitter, with a cumulative view count of 1.2 million. We believe that the CoVideo could play a useful role in improving COVID-19 hygiene uptake and mitigate the spread of this disease. However, it is unclear if the CoVideo improves behavioral intent toward COVID-19 hygiene.

We hypothesize that the CoVideo will be effective in improving behavioral intent toward COVID-19 hygiene when compared with an attention placebo control (APC) video or no video. The study results will inform the design of future E-E videos to disseminate evidence-based health recommendations related to COVID-19 as well as other public health emergencies.

## Objectives

The study aims to achieve the following objectives. To:

1. Quantify people’s interest in watching a short, animated video about COVID-19 hygiene (CoVideo).

2. Establish the CoVideo’s effectiveness in increasing behavioural intent toward COVID-19 hygiene.

3. Establish the CoVideo’s effectiveness in improving COVID-19 hygiene knowledge.

## Trial Design

The present study is a multi-site, parallel group, randomized controlled trial (RCT) comparing the effectiveness of the CoVideo against an APC video or no video. Randomization is at a 1:1:1 ratio for the three arms of the trial.

Nested within each arm of the trial is a list experiment. Participants will be randomized at a 1:1 ratio to a control or treatment group. The control group will receive a list of five items and the treatment group will receive the same five items plus a sensitive item. We use the list experiment to reduce response bias associated with socially desirable answers to COVID-19 questions.

The trial arms and components are shown in Figure 1.

## Methods: Participants, interventions and outcomes

### Study setting

This will be an online study setting. We will use the online recruitment platform Prolific Academic (ProA: https://www.prolific.co) to recruit participants from the United States, the United Kingdom, Germany, Spain, Mexico, and France. We will host and deploy our study on an online platform called Gorilla™ ([www.gorilla.sc](http://www.gorilla.sc)). Gorilla is a cloud platform that provides versatile tools to undertake online, experimental, and behavioral research.^8^

### Eligibility criteria

Registered participants on the ProA platform must be between the age of 18 and 59 years (male, female, or other) and have current residence in the United States, the United Kingdom, Germany, Spain, Mexico, or France. Participants will be excluded from the study if they cannot speak English, German, French, or Spanish (since the instructions and survey questions will be available only in these 4 languages).

Figure 1: Shows the trial design, which consists or three arms. Participants will be randomly assigned (at a 1:1:1 ratio) to the CoVideo (Arm 1), an APC video (Arm 2), or no video (Arm 3). Each arm has a list experiment, with participants split (at a 1:1 ratio) into a control or treatment group.

###
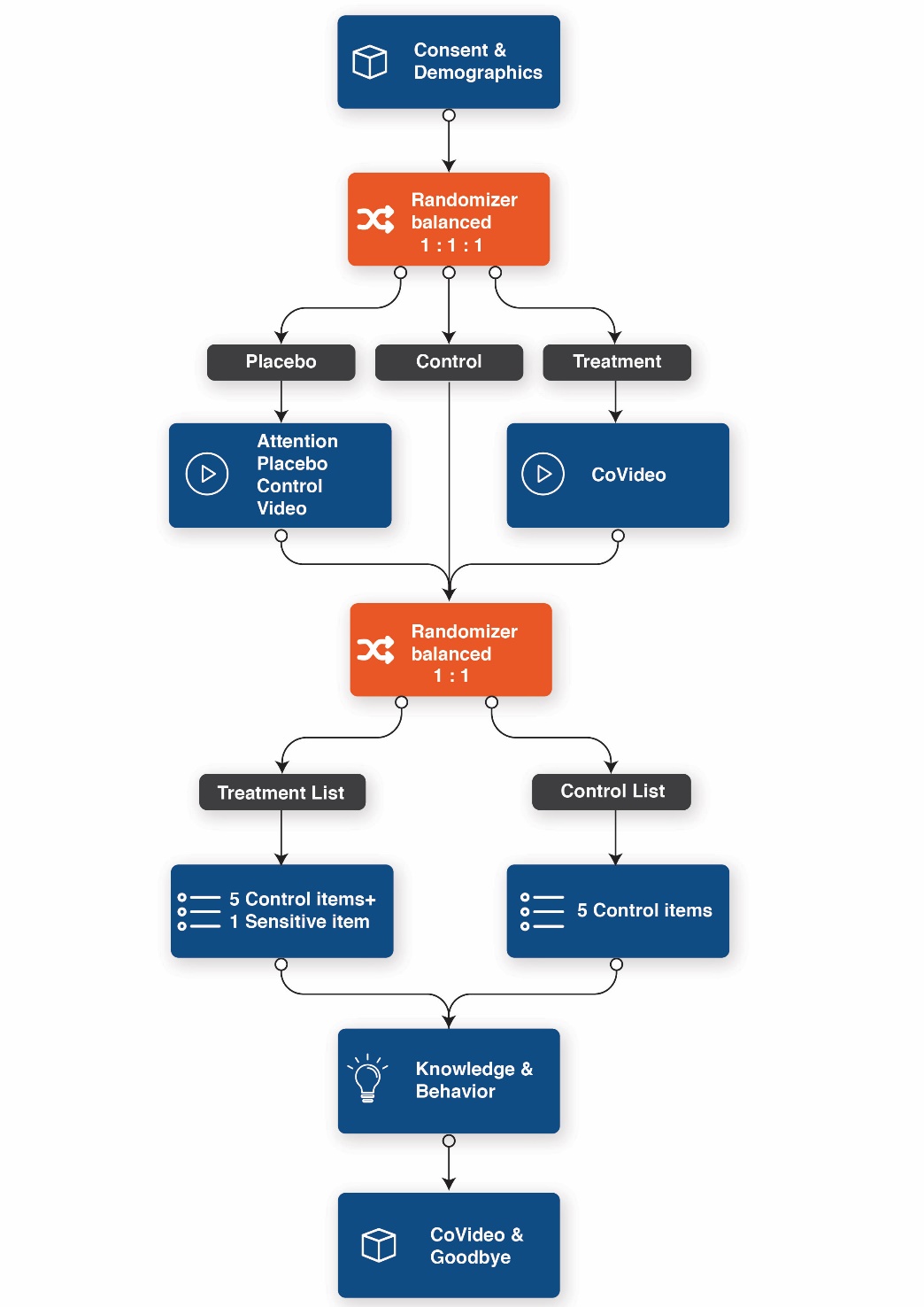


### Ethical approval

Ethical approval was obtained from the Stanford University IRB on April 12, 2020, protocol #55820.

### Who will take informed consent?

Participants will undergo a process of informed consent. The consent form will be hosted on the ProA platform. After selecting our study, participants will be shown the consent form. The form explains the purpose of the study, the risks and benefits of the research, and how to contact the study investigators (or Stanford University ethics review board) about problems experienced during the course of the study. By clicking the link, participants consent to participate in our study, and will be redirected to the Gorilla platform. On the landing page, there will be additional information about the Gorilla platform. Participants can exercise their freedom to participate (or not) at recruitment or at any point during the study.

## Interventions

### Intervention description

The intervention is an E-E video about COVID-19 hygiene (CoVideo). Developed by our co-author (MA) for Stanford Medicine, the CoVideo is animated with sound effects, and has no words, speech, or text. The CoVideo shows how the novel coronavirus is spread (airborne, physical contact) and recommends best hygiene practices to prevent onward transmission (staying at home, not congregating in public spaces, and sanitizing hands/surfaces). The CoVideo also covers the mass media coverage of the outbreak and the public’s response to this media coverage, which includes a subplot on the stockpiling of essential goods, and the impact thereof on health-care services and resources (e.g., doctors being unable to access protective equipment). The CoVideo, which can be viewed at <https://www.youtube.com/watch?v=rAj38E7vrS8>, was designed for universal reach and optimized for release on social media channels.

Each trial arm consists of three components: 1) a video intervention (the CoVideo, APC video, or no video), 2) a list experiment, and 3) survey questions about COVID-19 knowledge and media use. Participants will begin the online study by answering basic demographic questions about their age, sex, country of residence, and highest education completed. Thereafter, participants will be randomly assigned to one of three trial arms (see Figure 1). *Arm 1*: the CoVideo, list experiment, and questionnaire survey. *Arm 2*: the APC video, list experiment, and questionnaire survey, and CoVideo. *Arm 3*: list experiment, questionnaire survey, and CoVideo. After completing the list experiment and questionnaire survey, participants in Arms 2 and 3 will receive the CoVideo to ensure post-trial access to treatment.^9^

### Explanation for the choice of comparators

The comparators are an APC video (Arm 2) or no video (Arm 3). The APC video is similar in style to the CoVideo; it is also animated with a duration of 2.30 minutes, has sound effects but no words, speech, or text. The video message is about how small choices become actions, which become habits, which become a way of life. We have edited the video to remove the final frame which is in English. The video can be found at <https://www.youtube.com/watch?v=_HEnohs6yYw>.

We use these two comparators to measure the CoVideo’s effectiveness. 1) The CoVideo vs. APC, which measures the effect of video content without the attention effects. Drawing from psychotherapy efficacy studies, APC conditions should mimic the ‘inactive’ components of an intervention—such as the attention a patient receives from a therapist—while not containing any of the ‘active’ intervention components—such as the specific way the therapist conducts the treatment.^10^ 2) The CoVideo vs. do-nothing, which measures the total effect of the CoVideo. We do not make the assumption that the CoVideo is better than nothing (i.e., no video). It is possible that the CoVideo could motivate reactance to our public health message about adherence toward COVID-19 hygiene.^11–13^

In each arm, there will be a list experiment, with the control list as the comparator. The control list will include five statements about the participant’s intention to do activities that are not related to COVID-19 hygiene. The treatment list will include the same five control items and a statement about behavioral intent toward COVID-19 hygiene. The control list (comparator) is needed to measure the prevalence of behavioral intent toward COVID-19 hygiene. The list experiment is described in greater detail in the Outcomes section below.

### Criteria for discontinuing or modifying allocated interventions

We will not discontinue or modify the allocated interventions during the course of the study. Participants may choose to discontinue their participation on the Gorilla platform.

## Outcomes

This study will measure primary and secondary outcomes related to COVID-19 hygiene. By hygiene, we mean the adoption of behaviors or practices that reduce the chances of being infected or spreading COVID-19.^14^ In the CoVideo, we describe and promote five hygiene practices: i) social distancing or avoiding group gatherings, ii) frequently washing hands with soap and water or sanitizer, iii) cleaning surfaces at home (e.g., kitchen counters), iv) not sharing eating utensils, and v) avoidance of stockpiling essential goods (such as toilet paper and face masks).

As the primary outcome of this study, we aim to measure changes in behavioral intent toward COVID-19 hygiene. According to the Theory of Planned Behavior (TPB), the intention to act is considered the immediate determinant of action.^15^ Here, we frame behavioral intent as representing the participant’s commitment to undertake COVID-19 hygiene behaviors (or not) in the next week.^16,17^ As the secondary outcome of our study, we aim to measure changes in knowledge about COVID-19 hygiene. Knowledge is often considered to be a necessary but not sufficient condition for motivating a health behavior.^18^ Specifically, TPB posits that knowledge is more likely to be correlated with behavior if correct answers on the knowledge test support the practice of that behavior.^19^

*Primary outcome measure*

The primary outcome measure is the difference between the three trial arms in behavioral intent toward COVID-19 hygiene. To measure this difference, we will use a list randomization approach. The list experiment is designed to reduce response bias, since many participants will be primed to give socially desirable answers to COVID-19 hygiene questions.^20–22^ This approach will offer protection to participants who do not intend to adhere to COVID-19 hygiene recommendations without directly revealing this intention.

In each trial arm, participants will be randomly split (at a 1:1 ratio) into a control group or a treatment group. The control group will receive a list of five items. Participants will be asked to state how many of the items they agree with, without revealing which specific items they agree with. Here is one of the lists that the control group will receive:

***HOW MANY*** *of the five statements do you agree with? We don't want to know which ones, just answer* ***HOW MANY****.*

1. *Spend time watching TV*
2. *Do the vacuuming*
3. *Pick a fight with my partner*
4. *Eat a low sugar diet*
5. *Rinse my nose with salt water daily*

The treatment group will get the same list but with one additional item:

1. *Go out with my friends*

In list experiment terminology, this additional item is referred to as the ‘sensitive’ item. Here, the sensitive item captures agreement (or disagreement) to undertake the COVID-19 hygiene practice of social distancing. Asking the sensitive question in this indirect way may encourage the participant to answer the question truthfully, especially if the answer defies a COVID-19 recommendation (the social norm).^23^ As shown in Table 1, we present five list experiments that cover the topics of social distancing, washing hands, cleaning surfaces, sharing utensils, and stockpiling essential goods. Each trial arm will receive the same five list experiments. To avoid alerting the participant to the purpose of the list experiment, the items in each list will be randomly ordered. The five list experiments will also be presented in random order to avoid order effects. For each list, the minimum score is zero and the maximum score for the control list is five and six for the treatment list (there are five items in the control list and six items in the treatment list). We designed the items to minimize ceiling and floor effects.^22^ As described in the statistics section, we will use regression models to estimate the prevalence of each sensitive item.^23^ This data will be used to assess Objective 2.

After the list experiment, we will ask the participants in the control group to directly respond to the ‘sensitive’ item from each list experiment. Following from the above example, the corresponding direct question is:

*Do you Agree or Disagree with the following statement:* This week, I will go out with my friends.

The direct questions will enable us to quantify the magnitude of social desirability bias across the five sensitive items.

**Table 1: Primary outcomes.** In each trial arm, participants will be randomized (at a 1:1 ratio) to a control or treatment group. Both groups will receive five lists. For each list, the control group will get the first five items only; the treatment group will receive the five items and the sixth sensitive item, indicated by an asterisk (*). Each list experiment will be preceded by the question: “How many of the five/six statements do you agree with? We don't want to know which ones, just answer how many. This week I will…” To avoid order effects, the items in each list will be randomized as well as the presentation order of the lists. After the list experiments, the five sensitive items will be directly asked, and preceded by the statement: “Do you Agree or Disagree with the following statement: This week I will…”

| **List 1: Social distancing** | **List 2: Wash hands** |
| --- | --- |
| 1. Spend time watching TV | 1. Clip my toenails |
| 2. Do the vacuuming | 2. Spend time watching movies |
| 3. Pick a fight with my partner | 3. Clean the toilet |
| 4. Eat a low sugar diet | 4. Smoke marijuana |
| 5. Rinse my nose with salt water daily | 5. Eat fruit daily |
| *6. Go out with my friends** | *6. Wash my hands frequently** |
| **List 3: Clean surfaces** | **List 4: Share utensils** |
| 1. Watch a new TV series | 1. Spend time on the internet |
| 2. Spend time gardening by myself | 2. Do daily indoor exercises |
| 3. Try to go vegetarian | 3. Take an online course |
| 4. Have alcoholic drinks on at least three evenings | 4. Play a prank on my partner |
| 5. Catch up on last week's work | 5. Smoke cigarettes |
| *6. Clean kitchen counters after use** | *6. Clean my dishes after use** |
| **List 5: Stockpiling** | **Direct questions list** |
| 1. Spend time chatting with my friends online | 1. Go out with my friends |
| 2. Try new cooking recipes | 2. Wash my hands frequently |
| 3. Watch a pornographic movie | 3. Clean kitchen counters after use |
| 4. Clean all floor surfaces | 4. Clean my dishes after us |
| 5. Visit the World Health Organization (WHO) website | 5. Stock up on household supplies for a month |
| *6. Stock up on household supplies for a month** |  |

*Secondary outcome measure*

We will assess if the CoVideo improves knowledge about COVID-19 hygiene. There will be 20 True/False questions about the basic clinical aspects of COVID-19 and how the disease is spread. All participants will receive all 20 items. The knowledge items are shown in Table 2 and this data will be used to assess Objective 3.

We are also interested if the CoVideo elicits behavioral intent to access E-E media about health information. Again, we will use a list experiment because participants may feel socially ‘obliged’ to respond favourably to a question about E-E use after watching the CoVideo (and being compensated for participation in the online trial). The list experiment is shown in Table 2.

In Arms 2 and 3, we will offer the participants the choice to watch the CoVideo or end the survey. The Gorilla platform will record this response. If the ‘Watch Video’ button is clicked, Gorilla will record the time (in milliseconds) from the video start until the participant clicks the ‘Finish’ button (or until the video end at 2.30 minutes, whichever comes first). This data will be used to assess Objective 1.

### Participant timeline

Participants are expected to finish the trial (watch the video, complete list experiment, answer survey questions) in 10 minutes.

### Sample size

We calculated the sample size needed for pairwise comparisons between three groups using a one-way analysis of variance (ANOVA). The formula to calculate the sample size is:^24^

$$n_{A}=(\sigma_{A}^{2}+\sigma_{B}^{2}/\kappa)\left( \frac{Z_{1-\frac{\alpha}{\tau}}+Z_{1-\beta}}{\mu_{A}-\mu_{B}} \right)^{2}$$

where $\kappa=1$, which is the matching ratio, $\mu_{A}$ and $\mu_{B}$ are the group A and B means, $\sigma_{A}$ and $\sigma_{B}$ are the group A and B standard deviations, $\alpha=0.05$ is the Type-I error, $\beta=0.20$ is the Type-II error, $Z$ is the quantile function, and $\tau=2$ is the number of comparisons to be made. We assumed a mean of $\mu_{A}=2.0$ and $\mu_{B}=2.1$ for the control and treatment groups respectively. (In other words, we expect, on average, that the control group will agree with 2 out of the 5 items and the treatment group 2.1 of the 6 items.) We selected $\sigma_{A}=0.85$ and $\sigma_{B}$=0.95. This calculation gives a sample size of $n_{A}=n_{B}=n_{C}=1276$ for each group. For this study, we will recruit 20,100 participants, which exceeds the sample size required to determine an effect size of 0.1 with $\alpha=0.05$. A large sample size will be consistent with the study aim to measure the effectiveness of the CoVideo on a global scale.

### Recruitment

We will use the ProA platform to recruit the study participants. ProA has users from North America, Europe, and Latin America (currently, Mexico only). The user must open an account on ProA and provide his or her personal information. Participants must agree to ProA’s data privacy terms and conditions. ProA will assign each participant a unique, anonymized ID. The study investigators will also open an account on ProA. We will instruct the ProA platform on how many participants need to be recruited. ProA will filter out all participants who do not meet the eligibility criteria. Participant entry into our study will happen on a ‘first come, first served’ basis until the recruitment number (sample size) is reached. We will compensate the participants an equivalent of £1 for the expected 10 minutes completion time.

**Table 2: Secondary outcomes.** Shows the knowledge items, which require True/False responses. The list experiment for media use is also shown, which has the same procedure for the five list experiments described in Table 1. The question will be preceded by: “How many of the five/six statements do you agree with? We don't want to know which ones, just answer how many. This week, I will seek health information from...” The sensitive item is indicated by an asterisk (*). The items in the list will be randomly ordered.

| **COVID-19 knowledge items** | | **Media access to health information** |
| --- | --- | --- |
| The current coronavirus cannot be spread from person to person | Wash your hands frequently with soap and water | 1. Television news channels |
| The current coronavirus cannot survive on surfaces for more than a few minutes | Regularly rinse your nose with salt water | 2. Social media celebrities |
| Some people with COVID-19 infection may experience a cough | Avoid touching your face | 3. Religious leaders on the internet |
| Some people with COVID-19 infection do not experience a fever | Avoid shaking hands with other people | 4. Public health agency websites |
| The current coronavirus spreads from person to person through small droplets from the mouth | Avoid places that are crowded with people (like bars, restaurants or performances) | 5. Conversations with family and friends |
| The current coronavirus spreads from person to person through small droplets from the nose | Eat garlic with each meal | *6. Animated videos made by health experts** |
| You can catch COVID-19 by touching a contaminated surface and then touching your face | Avoid sharing eating utensils with others |  |
| Antibiotics can be used to treat COVID-19 infection | It is a good idea to buy a large supply of essential goods such as toilet paper to keep in your home |  |
| Cleaning surfaces with soap and water is an effective way to kill the current coronavirus | Wear a face mask even if you don't have COVID-19 symptoms |  |

## Assignment of interventions: allocation

The Gorilla platform is specifically designed to host and implement online experimental studies (implementation). Gorilla will randomly allocate participants to the intervention (CoVideo), placebo (APC), or control (no video) arm (sequence generation). Gorilla will use a web-based randomization algorithm, which is unknown to us (concealment mechanism).

## Assignment of interventions: Blinding

### Who will be blinded

Because ProA handles the interaction between the study investigators and participants, the participants will be completely anonymous to the study investigators. Only the participant’s unique, anonymized ID will be used to manage the linking between the ProA and Gorilla platforms. The outcome measures will be self-reported and submitted anonymously. The study investigators and those involved in the data analyses and statistics will be blinded to the group allocation.

## Data collection and management

### Plans for assessment and collection of outcomes

Data will be collected on the Gorilla platform. Participants will submit data by clicking on the response buttons. We expect to collect data from the 20,100 participants over a three to four week period.

### Plans to promote participant retention and complete follow-up

The expected completion time for the experiment is 10 minutes. Participants will be automatically timed-out of Gorilla if they take longer than 45 minutes to complete the survey. The time-out is to ensure that participants do not clog up the system with incomplete surveys. Since the participants are anonymous to us, there is no way to initiate follow-up in the maximum 45 minute time limit.

### Data management

All trial participants will be assigned a unique, anonymized string ID. The ID will be used on the Gorilla platform and linked to the participant’s responses. Gorilla will store the trial data on its cloud platform, hosted on Microsoft Azure in the Republic of Ireland. The Gorilla database is encrypted using industry-standard cryptography. The study investigators own the research data that has been collected using Gorilla and have complete control over it. The study investigators can generate and access the completely anonymized data from the Gorilla platform. The data will be downloaded and safely stored for statistical analysis on a computing system maintained by the University of Heidelberg in Germany.

### Confidentiality

The participants, who are completely anonymous to us, will have no identifying information associated with their unique IDs. We will inform participants that if they email the study investigators then their names could be revealed to us. The study investigators will keep this information confidential.

## Analysis

### Descriptive analysis

We will use descriptive statistics to obtain means and standard deviations of the demographic data (age, sex, country of residence, and education status), the knowledge items about COVID-19, and the direct questions about behavioral intent to take-up COVID-19 hygiene practices.

### Statistical methods for primary and secondary outcomes

We will use regression models to estimate the difference in the means of the intervention, placebo, and control groups. Blaire and Imai^23^ have developed regression estimators for the analysis of list data. Assuming that the response has a Poisson-Binomial distribution, they propose a nonlinear least-squares estimator that combines two possibly nonlinear regression models. If the model contains only an intercept, then the model reduces to a difference-in-means comparison. In a sensitivity analysis, we will use the proposed regression framework to adjust for the participants’ demographic characteristics. To measure bias, we will also use the same regression framework to estimate the mean difference in responses to the direct and indirect questions about behavioral intent toward COVID-19 hygiene. For the secondary outcome, we will leverage a modified Poisson regression framework to measure the difference in knowledge across the intervention, placebo, and control groups. We will use the statistical package *R* to undertake the analysis.

### Interim analyses

No interim analyses are planned.

### Methods for additional analyses (e.g. subgroup analyses)

We will conduct both country-specific and cross-country pooled analyses, provided sufficient sample sized can be recruited from each country.

### Methods in analysis to handle protocol non-adherence and any statistical methods to handle missing data

Participants will have a 30 second time limit to answer each knowledge item. This limit is to prevent participants from searching for answers on the internet. If the participant times out, they will receive a missing value of 9. This missing value will be recoded as an incorrect answer to the knowledge item, since the participant could not correctly answer the question in the allotted time.

### Plans to give access to the full protocol, participant level-data and statistical code

This document is the full protocol. Anyone interested in other data or documentation should contact the corresponding author.

## Oversight and monitoring

### Composition of the coordinating centre and trial steering committee

The trial will be overseen by a Trial Steering Committee (TSC). The TSC will have an independent chairperson and members but also includes the trial collaborators. Two TSC meetings are planned.

### Adverse event reporting and harms

Because the participants are anonymous to us, we will not be able to report any adverse events or harms. It is unlikely there will be adverse events given the format of our 10 minute, online trial. The auditing of trial conduct will be addressed in the two TSC meetings.

### Dissemination plans

We will disseminate the study findings through journal publications and conference presentations.

## Discussion

There is a critical need for public health actors to disseminate scientific information about the prevention and treatment of COVID-19. Our overall research agenda is to design E-E content that facilitates the dissemination of this information to a global audience at a rapid scale. To this end, we have produced a short, animated video (CoVideo) that provides information about the best practices for COVID-19 hygiene. Within the first week of release, the CoVideo went viral, highlighting its potential to inform the public on how to manage and reduce the spread of COVID-19.

Our study is an affordable, pragmatic trial to evaluate the effectiveness of the CoVideo on behavioral intent toward COVID-19 hygiene. To evaluate the effectiveness of the CoVideo, we leverage three innovative approaches.

First, we will use state-of-the-art online technology to implement our trial. The ProA platform will enable us to rapidly recruit a large number of participants who speak different languages and live in different countries in Europe, North America, and Latin America. This online recruitment resource will enable us to evaluate the effectiveness of the CoVideo across a diverse audience on a truly global scale.

Second, we will implement the study on the Gorilla platform, which is designed to host and facilitate experimental trials in behavioral research. The Gorilla platform will enable us to implement a multi-site, parallel group RCT with randomization at two different levels: 1) participants will be randomized to the intervention, placebo, or control arms; 2) within each arm, participants will then be randomized to receive a control list or the control list with a sensitive item. Within each list experiment, the list items will be randomly ordered. The list experiments will also be randomly ordered to avoid order effects. All randomization will be performed automatically by the Gorilla randomization algorithm, which demonstrates the platform’s potential to implement innovative trial designs and concepts.

Third, we will use a list experiment as the primary outcome of this study. Given the unprecedented nature of the COVID-19 outbreak, and the media attention it has received, we assume that participants will already be primed to give socially acceptable responses to questions about COVID-19 hygiene. In public, individuals will say they will adhere to social distancing recommendations. But in private, they may attend secret ‘corona parties’.^25^ The list experiment gets around the problem of eliciting untruthful answers to socially desirable questions. Further, we have designed the trial in a way, making full use of the Gorilla features, to measure the magnitude of social desirability bias toward COVID-19 hygiene questions.

We expect that our study will make important contributions to the E-E literature. Importantly, lessons learned can help us to improve the design of E-E videos to disseminate public health information for future pandemics. And we may hopefully demonstrate that our CoVideo, which has been viewed more than a million times on social media thus far, will have contributed to current public health efforts to keep people safe and reduce the spread of COVID-19. Our results will also guide future E-E strategies to support the long-term COVID-19 response, as countries are easing lock-down restrictions.

**References**

1 Cinelli M, Quattrociocchi W, Galeazzi A, *et al.* The COVID-19 Social Media Infodemic. *arXiv* 2020; published online March 10.

2 Mian A, Khan S. Coronavirus: the spread of misinformation. *BMC Med* 2020; **18**: 89.

3 Kouzy R, Abi Jaoude J, Kraitem A, *et al.* Coronavirus Goes Viral: Quantifying the COVID-19 Misinformation Epidemic on Twitter. *Cureus* 2020; published online March 13. DOI:10.7759/cureus.7255.

4 Garfin DR, Silver RC, Holman EA. The novel coronavirus (COVID-2019) outbreak: Amplification of public health consequences by media exposure. *Heal Psychol* 2020; **In Press**. DOI:10.1037/hea0000875.

5 Garrett L. COVID-19: the medium is the message. *Lancet* 2020; **395**: 942–3.

6 Zhao Z, Zhu Y-Z, Xu J-W, *et al.* A mathematical model for estimating the age-specific transmissibility of a novel coronavirus. *medRxiv* 2020; : 2020.03.05.20031849.

7 Hahn U, Lagnado D, Lewandowsky S, Chater N. Crisis knowledge management: Reconfiguring the behavioural science community for rapid responding in the Covid-19 crisis. *psyarxiv*.

8 Anwyl-Irvine AL, Massonnié J, Flitton A, Kirkham N, Evershed JK. Gorilla in our midst: An online behavioral experiment builder. *Behav Res Methods* 2020; **52**: 388–407.

9 Doval D, Shirali R, Sinha R. Post-trial access to treatment for patients participating in clinical trials. *Perspect Clin Res* 2015; **6**: 82.

10 Freedland KE, Mohr DC, Davidson KW, Schwartz JE. Usual and unusual care: Existing practice control groups in randomized controlled trials of behavioral interventions. *Psychosom Med* 2011; **73**: 323–35.

11 Dillard JP, Shen L. On the nature of reactance and its role in persuasive health communication. *Commun Monogr* 2005; **72**: 144–68.

12 Miller CH, Lane LT, Deatrick LM, Young AM, Potts KA. Psychological Reactance and Promotional Health Messages: The Effects of Controlling Language, Lexical Concreteness, and the Restoration of Freedom. *Hum Commun Res* 2007; **33**: 219–40.

13 Richards AS, Banas JA. Inoculating Against Reactance to Persuasive Health Messages. *Health Commun* 2015; **30**: 451–60.

14 WHO. Coronavirus disease (COVID-19) advice for the public. World Heal. Organ. 2020. https://www.who.int/emergencies/diseases/novel-coronavirus-2019/advice-for-public.

15 Ajzen I. The theory of planned behavior. *Organ Behav Hum Decis Process* 1991; **50**: 179–211.

16 Lin N, Roberts KR. Predicting and explaining behavioral intention and hand sanitizer use among US Army soldiers. *Am J Infect Control* 2017; **45**: 396–400.

17 Connor M, Sparks P. The theory of planned behavior and reasoned action approach. In: Connor M, Norman P, eds. Predicting and changing health behavior: Research and practice with social cognition models, 3rd edn. Maidenhead: Open University Press, 2015: 142–88.

18 Helweg-Larsen M, Collins B. A social psychological perspective on the role of knowledge about AIDS in AIDS prevention. *Curr Dir Psychol Sci* 1997; **6**.

19 Ajzen I, Joyce N, Sheikh S, Cote NG. Knowledge and the Prediction of Behavior: The Role of Information Accuracy in the Theory of Planned Behavior. *Basic Appl Soc Psych* 2011; **33**: 101–17.

20 Kramon E, Weghorst K. (Mis)Measuring Sensitive Attitudes with the List Experiment. *Public Opin Q* 2019; **83**: 236–63.

21 Corstange D. Sensitive Questions, Truthful Answers? Modeling the List Experiment with LISTIT. *Polit Anal* 2009; **17**: 45–63.

22 Glynn AN. What Can We Learn with Statistical Truth Serum? *Public Opin Q* 2013; **77**: 159–72.

23 Blair G, Imai K. Statistical Analysis of List Experiments. *Polit Anal* 2012; **20**: 47–77.

24 Rosner B. Fundamentals of Biostatistics, 7th edn. Boston: Brooks/Cole, 2010.

25 Levine J. New Yorkers are throwing ‘corona potlucks’ and visiting ‘speakeasies’. *New York Post* 2020.
